# Supplementary material for: Discovering the Active Ingredients of Medicine and Food Homologous Substances for Inhibiting the Cyclooxygenase-2 Metabolic Pathway by Machine Learning Algorithms
Source: Molecules. 2023 Sep 23;28(19):6782. doi: 10.3390/molecules28196782 (PMC10574661; doi:10.3390/molecules28196782)
Supplement: Supplementary file 1 [file molecules-28-06782-s001.zip › FigureS1.pdf]

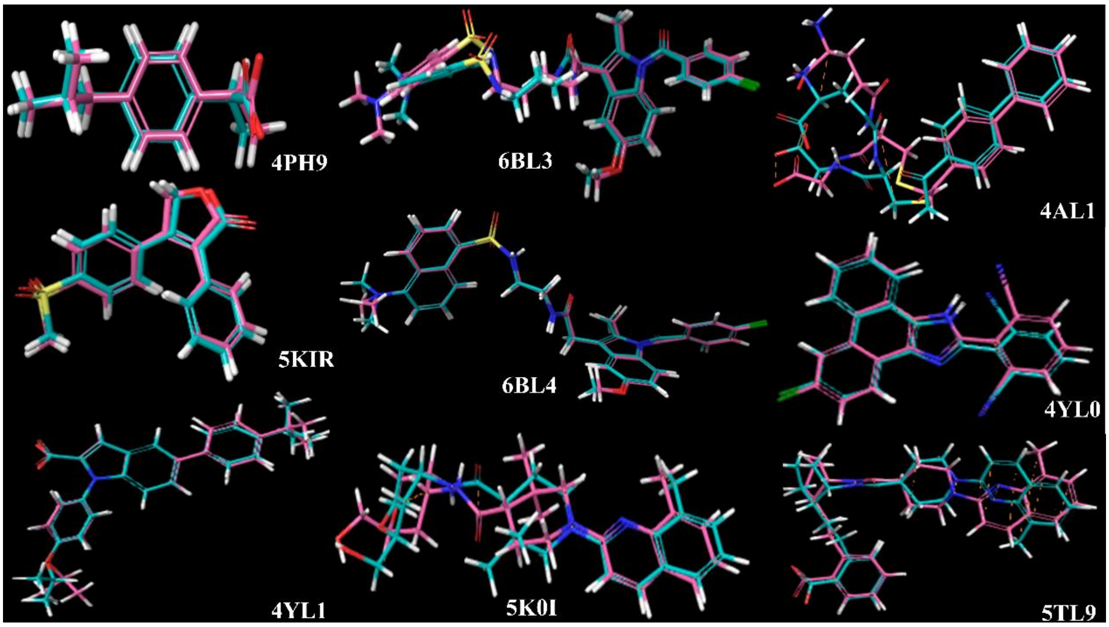

Figure S1. The alignment of docking conformations (blue) and crystal conformations (pink) of the ligand in nine PDB crystal structures used in this work, PDB ID were labeled at the bottom of each figure.
