# Supplementary material for: Discovering the Active Ingredients of Medicine and Food Homologous Substances for Inhibiting the Cyclooxygenase-2 Metabolic Pathway by Machine Learning Algorithms
Source: Molecules. 2023 Sep 23;28(19):6782. doi: 10.3390/molecules28196782 (PMC10574661; doi:10.3390/molecules28196782)
Supplement: Supplementary file 1 [file molecules-28-06782-s001.zip › Table S10.pdf]

Table S10. Selection of PDB crystal complex structures for docking with screened medicine and food homologous (MFH) candidates.

Molecular structures	Molecules	PDB	Protein
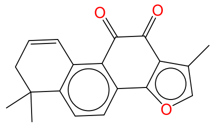	cmp_A1	5KIR	COX2
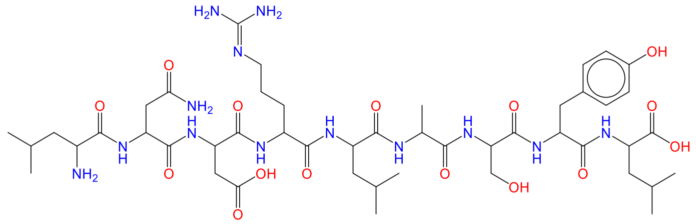	cmp_A2	6BL3	COX2
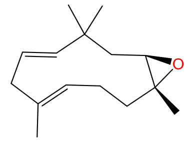	cmp_A3	4PH9	COX2
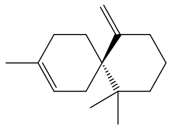	cmp_A4	4PH9	COX2
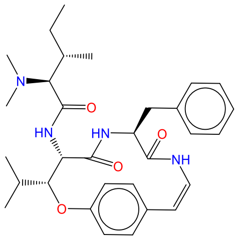	cmp_A5	6BL4	COX2
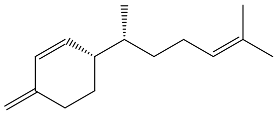	cmp_A6	4PH9	COX2
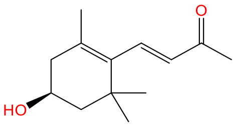	cmp_A7	4PH9	COX2
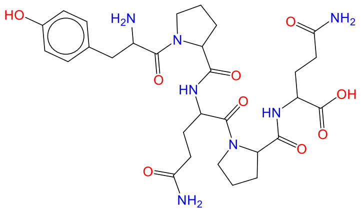	cmp_A8	6BL4	COX2

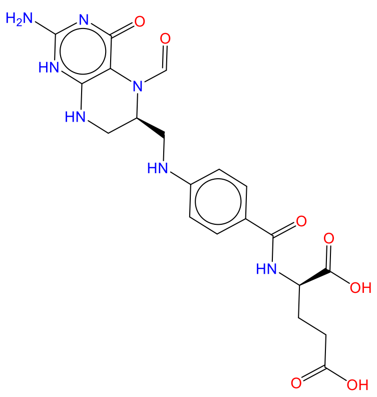	cmp_A9	6BL4	COX2
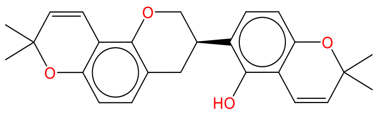	cmp_A10	6BL3	COX2
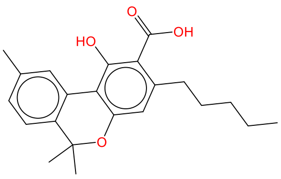	cmp_B1	4YL0	mPGES1
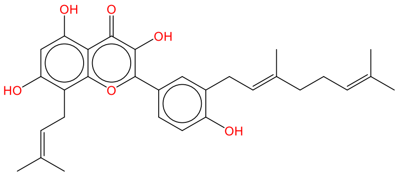	cmp_B2	4YL1	mPGES1
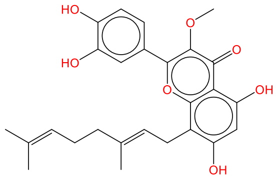	cmp_B3	4YL1	mPGES1
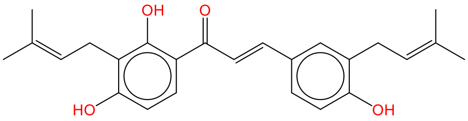	cmp_B4	4AL1	mPGES1
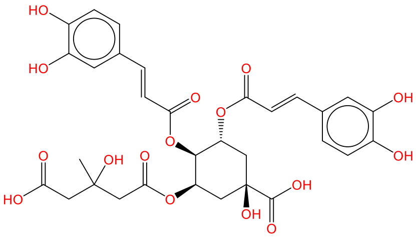	cmp_B5	4AL1	mPGES1
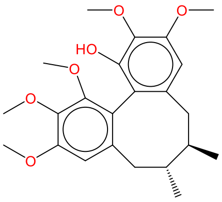	cmp_B6	4YL0	mPGES1

cmp_B7 4YL0 mPGES1

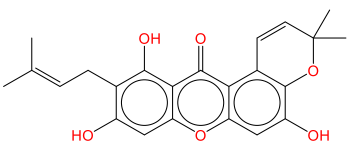

cmp_B8 5TL9 mPGES1

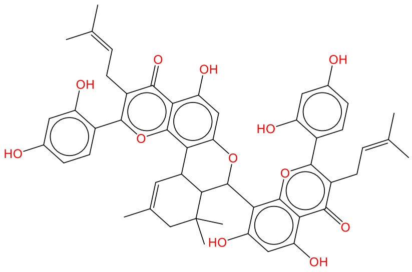

cmp_B9 5TL9 mPGES1

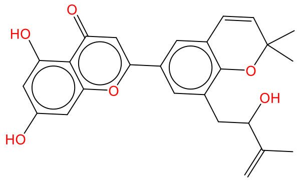

cmp_B10 5K0I mPGES1

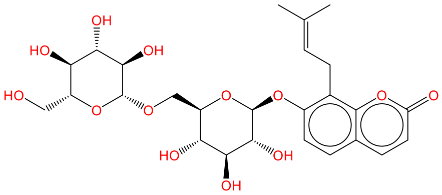

cmp_B11 4YL0 mPGES1

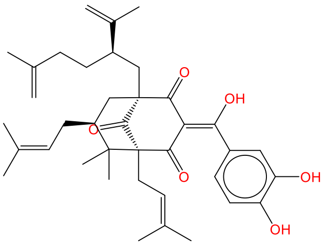

cmp_B12 4YL0 mPGES1

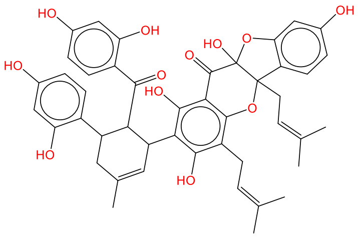

cmp_B13 4YL0 mPGES1

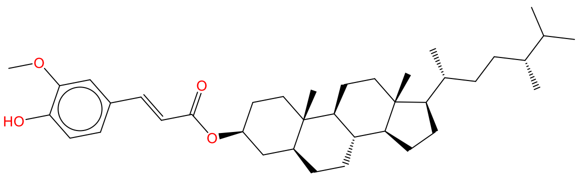

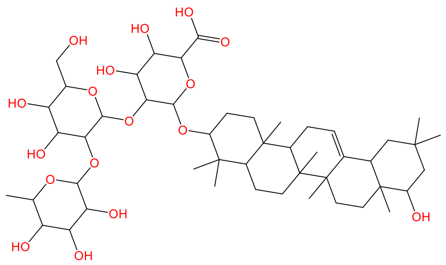	cmp_B14	4YL0	mPGES1
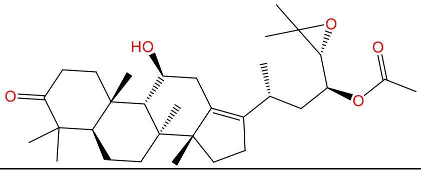	cmp_B15	4YL0	mPGES1
